# Comparison of magnetic resonance‐guided laser interstitial thermal therapy corpus callosum ablation to open microsurgical corpus callosotomy: A single‐center retrospective cohort study

**DOI:** 10.1002/epi4.12835

**Published:** 2023-11-10

**Authors:** H. Westley Phillips, Jasmine L. Hect, Emily Harford, Evelyn Pan, Taylor J. Abel

**Affiliations:** ^1^ Department of Neurosurgery Stanford University School of Medicine Palo Alto California USA; ^2^ Department of Neurological Surgery University of Pittsburgh Pittsburgh Pennsylvania USA; ^3^ Department of Bioengineering University of Pittsburgh Pittsburgh Pennsylvania USA

**Keywords:** atonic seizures, laser ablation, pediatric drug‐resistant epilepsy, seizure outcomes

## Abstract

**Objective:**

Corpus callosotomy (CC) is an important treatment for atonic seizures in patients with generalized or multifocal drug‐resistant epilepsy (DRE). Traditionally, CC is performed via an open microsurgical approach, but more recently, MR‐guided stereotactic laser interstitial thermal therapy (LITT) corpus callosum ablation (CCA) has been developed to leverage the safety and minimally invasive nature of LITT. Given the recent adoption of CCA at select centers, how CCA compares to CC is unknown. We aim to compare the clinical seizure outcomes of CCA and CC after extended follow‐up.

**Methods:**

We performed a retrospective cohort study to compare the effectiveness and safety of CC to CCA from 1994 to 2022. The primary outcome was a 50% reduction in target seizure. Secondary outcome measures were postoperative length of stay, adverse events, and other effectiveness metrics. Comparative statistics were executed using Stata. Normality for continuous variables was assessed, and parametric statistics were utilized as needed. Frequency was compared with chi‐squared or Fischer's exact tests, when applicable.

**Results:**

Data from 47 operations performed on 36 patients were included in this study, of which 13 (36%) patients underwent 17 CCA. Patients who received CCA had similar rates of meaningful reduction (>50%) of atonic seizures as their CC counterparts (55% vs 70% *P* = 0.15). Patients undergoing CCA had significantly shorter hospitalizations than those receiving CC (2.5 vs 6.0 days *P* < 0.001). There was no significant difference in rates of postoperative complications between the groups, although the magnitude of the complication rates was lower in the CCA cohort (12% vs 28%).

**Significance:**

This early experience suggests CCA has similar outcomes to traditional CC, albeit with a shorter hospital stay. However, future studies are necessary to investigate the noninferiority between these two approaches. Large multicenter studies are necessary to investigate differences in adverse events and whether these findings generalize across other centers.

**Key points**

- Corpus callosotomy (CC) is a safe and effective treatment for atonic seizures.
- Laser interstitial thermal therapy corpus callosum ablation (CCA) provides a safe and effective alternative to traditional open CC.
- CCA is associated with similar clinical outcomes compared to CC while having shorter hospital stays.

## 1 INTRODUCTION

Corpus callosotomy (CC) is an important option for palliative treatment of multifocal and generalized drug‐resistant epilepsy (DRE), especially in cases where atonic or tonic seizures are the predominant semiology.^1^, ^2^, ^3^ Traditionally, CC is performed via open craniotomy, utilizing the interhemispheric approach for microdissection of the corpus callosum. Despite its effectiveness, many patients are not considered candidates for CC given the morbidity of open cranial surgery. Further, the idea of cranial surgery to achieve improved quality of life or functionality is a major barrier for many patients and caregivers. Recent adoption of MR‐guided stereotactic laser interstitial thermal therapy (LITT) in the treatment of other neurosurgical disorders has resulted in the emergence of corpus callosum ablation (CCA) as a viable and effective treatment option for DRE, effectively expanding the cohort of potential operative candidates.^4^, ^5^, ^6^, ^7^

While this procedure is often referred to as a variant of the traditional CC in the contemporary literature, the etymology of “‐otomy,” a derivative of the Greek suffix ‐tómos, meaning to sharply cut or separate, suggests ablative procedures of the corpus callosum for the treatment of DRE may have distinct mechanisms of action compared to traditional CC. For this reason, we will refer to LITT‐mediated ablation of the corpus callosum as CCA with an understanding that the current terminology, including LITT‐CC, has been readily accepted throughout the literature despite this discrepancy.

Unlike traditional CC, CCA is lauded for its minimally invasive approach, decreased blood loss, and operative time, in addition to decreased hospitalization and rates of complications.^5^, ^8^, ^9^ Several studies have reported the safety and effectiveness of CCA compared to open CC with short‐interval follow‐up, but few studies have compared the durability of CCA outcomes over time. We therefore present this study outlining our experience with CCA and comparing surgical variables and seizure outcomes to CC, with follow‐up periods extending beyond 1 year. A primary outcome of 50% seizure reduction of target seizure and secondary outcomes of length of hospital stay and rates of complications were assessed as surrogates of safety and effectiveness.

## 2 METHODS

### 2.1 Patient selection

A single‐institution retrospective cohort study of pediatric patients undergoing CCA and CC for DRE at the Children's Hospital of Pittsburgh from 1994 to 2022 was performed. Approval from the Institutional Review Board (IRB) was obtained for retrospective data collection and analysis of epilepsy patients using institutional database #20070281. All patients were referred for callosotomy or callosal ablation by an expert panel who determined the patient suffered from drug‐resistant atonic or tonic seizures. The decision to target the anterior two‐thirds versus the entire corpus callosum was made by an expert panel and was based on the patient's preoperative functional and cognitive status. Anterior two‐thirds callosotomy or callosum ablation included division of the corpus callosum from the genu to the posterior body of the callosum with preservation of the splenium.

Data were collected in a retrospective fashion using the epilepsy surgery database. Patient demographics, operative details, postoperative course, and seizure outcomes were collected. Seizure characteristics were collected from the primary epileptologist's clinical and follow‐up documentation. The primary outcome was a 50% reduction in atonic seizures at 1‐year follow‐up. The secondary outcomes included other measures of effectiveness, complications, and hospital length of stay. Complications were stratified into groups based on characteristics, time of complication, and, secondarily, their necessity for operative intervention.

### 2.2 Statistical analysis

Comparative statistics were executed using Stata, version 16.0 (StataCorp. 2019. *Stata Statistical Software: Release 16*. College Station, TX: StataCorp LLC). Normality for continuous variables was assessed, and parametric statistics were utilized as needed. A *t* test was performed to compare continuous variables with approximately normal distributions, and the mean (SD) is reported. Wilcoxon rank‐sum was used to compare continuous, non‐normally distributed variables, and the median (25%–75% IQR) is reported. Frequency is reported as the percentage of the total and was compared using chi‐squared or Fisher's exact tests when sample sizes were less than 5 in any comparison group. Outcomes were compared for patients with at least 1 year of follow‐up (n = 5 CCA vs n = 19 CC).

## 3 RESULTS

**FIGURE 1:**
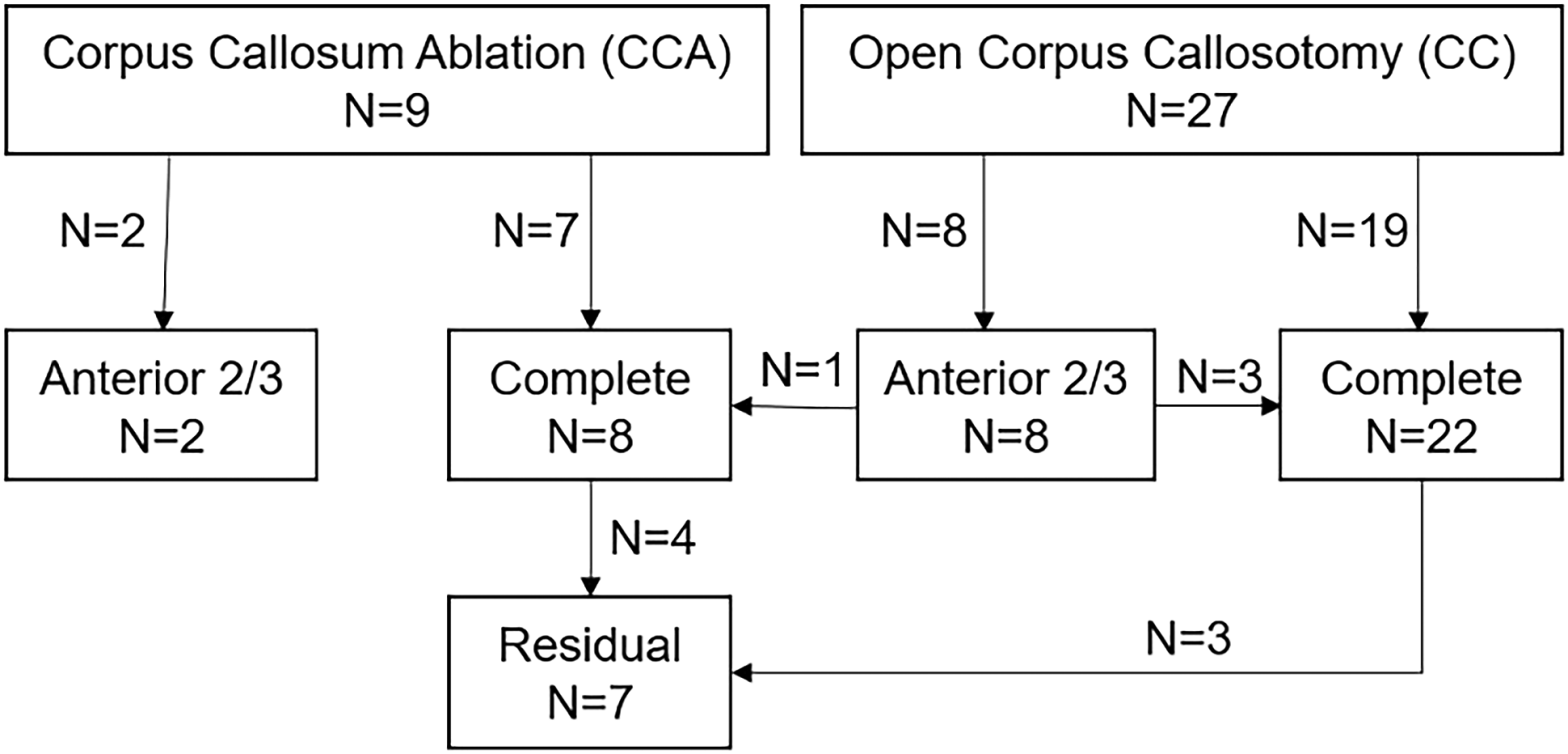
Flowchart demonstrating approach employed for 36 patients included in this study.

We identified 36 patients who underwent 47 procedures, of which 13 patients underwent 17 CCA procedures (Table 1, Figure 1). All patients had sufficient data for inclusion in the study. There were no significant differences in the demographic features of the CCA versus CC cohorts. Demographic variables were compared between CCA (N = 9) and CC (N = 27) at the time of their first intervention, whereas surgical variables were compared across all interventions (CCA = 17 and CC = 30). Four patients underwent open CC as a primary intervention and crossed over to CCA as a secondary intervention for either completion of partial anterior 2/3 (N = 1) or ablation of residual white matter (N = 3). These patients were not included in seizure outcome comparisons. The length of follow‐up was calculated from the first intervention in the event the patient underwent repeat operations. The CC cohort encompassed open surgical interventions spanning from 1994 until 02/2022. The first CCA was performed 1/2020 and the last patient with adequate follow‐up received a CCA 07/2022 (Table S1). The complete profile of both cohorts is outlined in Table 1. Of note, the median age (25%–75% IQR) for CCA and CC were 13^10^, ^11^, ^12^, ^13^, ^14^, ^15^, ^16^ and 12^8^, ^9^, ^10^, ^11^, ^12^, ^13^, ^14^, ^15^ years of age, respectively. While virtually all patients presented with clinical features of Lennox‐Gastaut Syndrome (LGS) and could be considered “LGS‐like,” a total of 41% of patients in the CC cohort and 33% of patients in the CCA group had a confirmed diagnosis of LGS. The most common presenting seizure type in both groups was infantile spasm, with 33% and 30% in the CCA and CC cohorts, respectively. The median (25%–75% IQR) follow‐up for the CCA cohort was 14.14 months (3.49–16.57) versus 44.94 (29.26–71.18) months in their CC counterparts. Of note, six of nine patients (66.67%) in the CCA cohort and 24 of 27 patients (88.89%) in the CC cohort had at least 6 months of follow‐up at the time of data analysis. Five of nine patients (55.56%) in the CCA cohort and 24 of 27 patients (88.89%) in the CC cohort had at least 1 year of follow‐up.

**TABLE 1:** Patient and surgical characteristics between callosum ablation and open callosotomy.

Primary intervention	Total N = 36	CCA N = 9	CC N = 27	*P*‐value
Age at first intervention (years)	12 (9–17)	13 (10–17)	12 (8–16)	0.51
Sex (female)	18 (50%)	6 (67%)	12 (44%)	0.44
Race (white)	34 (97%)	8 (89%)	26 (100%)	0.26
LGS	14 (39%)	3 (33%)	11 (41%)	1.00
Infantile spasms	11 (31%)	3 (33%)	8 (30%)	1.00
Extent of primary callosotomy				
Partial, anterior 2/3	10 (28%)	2 (22%)	8 (30%)	0.67
Complete callosotomy	26 (72%)	7 (78%)	19 (70%)	

All procedures	Total N = 47	CCA N = 17	CC N = 30	*P*‐value
Secondary callosotomy				
Completion of prior partial	4 (9%)	1 (6%)	3 (10%)	1.00
Disconnection of residual fibers	7 (15%)	7 (41%)	0 (0%)	0.001
Surgery length (h)^a^	6.17 (5.25–7.1)	7.42 (5.73–8.03)	6.025 (5.025–6.77)	0.36
Prior VNS	32 (68%)	8 (47%)	24 (80%)	0.027
VNS same OR	6 (13%)	4 (24%)	2 (7%)	0.17

^a^ Surgery length calculated for callosotomy only, excluding procedure length involving concomitant VNS or RNS.

There were no significant differences in operative time between the two groups (Table 1). However, the observed operative times in the CC cohort were lower than those in the CCA cohort, with median surgical times (25%–75% IQR) of 6.03 h (5.03‐6.77) and 7.42 h (5.73–8.03), respectively. There were significantly more patients in the CC cohort who had a prior vagus nerve stimulator (VNS) implantation than their CCA counterparts (80% vs 47% *P* = 0.027). However, there were no significant differences between groups in the requirement for adjuvant epilepsy surgical interventions for refractory seizures, including future LITT ablation, resective surgery, responsive neurostimulation (RNS), or VNS placement.

Seizure outcomes for both cohorts are outlined in Table 2. Four patients were excluded from seizure outcome comparisons in the case they had open CC as a primary intervention and crossed over to CCA as a secondary intervention for either completion of partial anterior 2/3 (N = 1) or ablation of residual white matter (N = 3). Of the remaining patients, five in the CCA cohort had at least 1 year of follow‐up versus 20 patients in the CC group, after excluding the four CC patients who underwent subsequent CCA for residual ablation. Across the entire treatment cohort, two patients (7%) attained complete seizure freedom at 1‐year postoperatively, and there was no significant difference in Engle I outcomes found between the CCA (0%) and CC (10%) cohorts, *P* = 0.680.

**TABLE 2:** Seizure outcomes (most recent intervention only).

	Total N = 32^a^	CCA N = 6	CC N = 26	*P*‐value
Complete seizure freedom with medication	2 (6%)	0 (0%)	2 (8%)	1.00
Transient postoperative seizure increase	7 (24%)	3 (50%)	4 (17%)	0.13
Number of ASMs at 1 y		N = 5	N = 18	1.00
Unchanged	15 (65%)	4 (80%)	11 (61%)	
Decreased	5 (22%)	1 (20%)	4 (22%)	
Increased	3 (13%)	0 (0%)	3 (17%)	
Atonic seizures at 1 y follow‐up		N = 5	N = 20	0.025
No improvement	8 (32%)	3 (60%)	5 (25%)	
Significant decrease (>50%)	12 (48%)	0 (0%)	12 (60%)	
No atonic seizures	5 (20%)	2 (40%)	3 (15%)	
Tonic seizures at 1 y follow‐up		N = 1	N = 13	0.43
No change	3 (21%)	1 (100%)	2 (15%)	
Significant decrease (>50%)	8 (57%)	0 (0%)	8 (62%)	
Resolved	1 (7%)	0 (0%)	1 (8%)	
Increased	2 (14%)	0 (0%)	2 (15%)	
Tonic–clonic seizures at 1 y follow‐up		N = 2	N = 12	
No change	8 (57%)	0 (0%)	8 (67%)	0.055
Significant decrease (>50%)	5 (36%)	1 (50%)	4 (33%)	
Resolved	0 (0%)	0 (0%)	0 (0%)	
Increased	1 (9%)	1 (50%)	0 (0%)	
Engel Score at 1 y follow‐up		N = 5	N = 20	0.68
Engel I	2 (8%)	0 (0%)	2 (10%)	
Engel II	3 (12%)	1 (20%)	2 (10%)	
Engel III	13 (52%)	2 (40%)	11 (55%)	
Engel IV	7 (28%)	2 (40%)	5 (25%)	
Months follow‐up	34.7 (16.3–63.6)	15.4 (11.4–21.5)	44.0 (29.3–71.2)	0.006
Future epilepsy surgery	11 (34%)	2 (33%)	9 (35%)	1.00
RNS	2 (6%)	1 (17%)	1 (4%)	0.34
Resection	1 (3%)	1 (17%)	0 (0%)	0.19
VNS	3 (9%)	2 (33%)	1 (4%)	0.083
Number of ASMS at last follow‐up		N = 6	N = 21	0.045
Unchanged	11 (41%)	5 (83%)	6 (29%)	
Decreased	6 (22%)	1 (17%)	5 (24%)	
Increased	10 (37%)	0 (0%)	10 (48%)	
Atonic seizures last follow‐up		N = 5	N = 22	0.15
No improvement	9 (33%)	3 (60%)	6 (27%)	
Significant decrease (>50%)	9 (33%)	0 (0%)	9 (41%)	
No atonic seizures	9 (33%)	2 (40%)	7 (32%)	
Tonic seizures at last follow‐up		N = 1	N = 17	0.50
No change	6 (33%)	1 (100%)	5 (29%)	
Significant decrease (>50%)	9 (50%)	0 (0%)	9 (53%)	
Resolved	1 (6%)	0 (0%)	1 (6%)	
Increased	2 (11%)	0 (0%)	2 (12%)	
Tonic–clonic seizures at last follow‐up		N = 2	N = 12	1.00
No change	9 (56%)	1 (50%)	8 (57%)	
Significant decrease (>50%)	7 (44%)	1 (50%)	6 (43%)	
Resolved	0 (0%)	0 (0%)	0 (0%)	
Increased	0 (0%)	0 (0%)	0 (0%)	
Engel Score at last follow‐up		N = 6	N = 24	0.39
Engel I	2 (7%)	0 (0%)	2 (8%)	
Engel II	3 (10%)	1 (17%)	2 (8%)	
Engel III	12 (40%)	1 (17%)	11 (46%)	
Engel IV	13 (43%)	4 (67%)	9 (38%)	

^a^ Four patients were excluded from CC cohort for follow‐up analysis because their most recent procedure was CCA for residual disconnection.

A transient increase in seizure frequency in the first 30 postoperative days was seen in both groups, with similar rates of 50% (CCA) and 17% (CC), respectively (*P* = 0.13). Of patients with at least 1 year of follow‐up in either cohort, 68% (n = 17) were noted to have a 50% or greater decrease in atonic seizures. There was a significant difference in improvement of atonic seizures at 1‐year follow‐up in the CCA cohort, with 40% (N = 2) having no atonic seizures at 1‐year follow‐up in the CCA cohort compared to 15% (N = 3) of patients being free of atonic seizures in the CC group (*P* = 0.025); however, this result must be interpreted with caution given the discrepancy of the respective sample sizes (N = 5 vs N = 20). While four CCA patients underwent callosotomy within the last year and are awaiting 1‐year follow‐up, no significant difference in atonic seizure eradication was observed when the time point was adjusted to last follow‐up (40% vs 32%, *P* = 0.31), although the magnitude of seizure freedom was greater in the CCA group. Similarly, there was no statistical difference in greater than 50% atonic seizure reduction when the time point of last follow‐up was analyzed, with 40% (N = 2) of patients in the CCA demonstrating at least >50% reduction as compared to 73% (N = 16) in the CC cohort. For the subset of patients that experienced preoperative tonic seizures (N = 18), rates of significant reduction were similar in both the CCA and CC cohorts at last follow‐up, although only one patient with CCA is included in this analysis, limiting generalizability. There was also no significant difference in generalized tonic–clonic (GTC) seizure reduction between groups at any time point. Fourteen percent of patients (N = 2) in the CC cohort demonstrated an increase in tonic seizures at 1 year, and 7% (N = 1) in the CCA group experienced an increase in tonic–clonic seizure activity.

Engel outcomes were also assessed at 1‐year follow‐up and longest follow‐up, which did not demonstrate any significant difference between the CCA and CC cohorts (*P* = 0.68 and 0.39). In the CCA group, 20% (N = 1) of patients were noted to have a good overall seizure outcome as denoted by Engel class I or II, versus 20% (N = 4) in the CC group at 1‐year follow‐up. While the majority of patients in this study remained on the same total number of antiseizure medications (ASMs) following surgery (65%), regardless of intervention, similar rates of ASM reduction were observed in the CCA (20%) and CC (22%) cohorts. Of note, three of four patients who underwent open CC as a primary intervention and CCA as a secondary intervention (completion of anterior 2/3, N = 1, and ablation of residual white matter, N = 3) had a 12‐month follow‐up (median 16.54 months, 25%–75% IQR 13.74–29.59). Atonic seizures were found to persist in 67% (N = 2) of cases and resolve in 33% (N = 1) at 12‐month follow‐up, and similarly, 33% were Engel III (N = 1) and 67% Engel IV. There were no changes in ASMs at follow‐up for this subset of patients.

Patients undergoing CCA had significantly fewer postoperative days in the hospital when compared to CC, with a median (25%–75% IQR) total length of stay of 2.5 (1.5–3.5 days) and 6 (4–8 days) days (*P* < 0.001), respectively (Table 3). This difference was likely due to shorter periods on the general ward, which were significantly lower for the CCA cohort (median 1 day) than the CC cohort (median 4 days) (*P* < 0.001). There was no significant difference in the length of stay in the intensive care unit or discharges to inpatient rehabilitation centers between groups (*P* = 0.61). There was also no significant difference in total complications between the two cohorts (Table 3). However, the amplitude of the rate of complications was lower in the CCA cohort compared to the CC (12% vs 23%, *P* = 0.45). Both cohorts had similar 30‐day readmission rates (CCA 0% vs CC 4%, *P* = 1.00). Complications requiring operative intervention or resulting in permanent neurologic deficits were invariably rare in both groups, and there was no significant difference appreciated (*P* = 1.00). Interestingly, there were no cases of hydrocephalus in either group.

**TABLE 3:** Postoperative complications and Hospitalization Outcomes (all procedures).

	Total N = 47	CCA N = 17	CC N = 30	*P*‐value
Complications				
Hemorrhage	8 (17%)	2 (12%)	6 (20%)	0.69
IVH	5 (11%)	1 (6%)	4 (13%)	0.64
EAH	5 (11%)	1 (6%)	4 (13%)	0.64
Infection	1 (2%)	0 (0%)	1 (4%)	1.00
CSF Leak	1 (2%)	0 (0%)	1 (4%)	1.00
Hydrocephalus	0 (0%)	0 (0%)	0 (0%)	
Complications requiring OR	1 (2%)	0 (0%)	1 (4%)	1.00
Readmit 30 days	5 (11%)	0 (0%)	5 (19%)	0.14
Hospitalization				
ICU (days)	1.5 (1–3)	1 (1–2.5)	2 (1–3)	0.61
Floor (days)	2 (1–4)	1 (0–1)	4 (2–6)	<0.001
LOS total (days)	4 (2–6)	2.5 (1.5–3.5)	6 (4–8)	<0.001
Discharge to rehab	9 (21%)	3 (18%)	6 (24%)	0.72
Transient neurologic deficit	16 (36%)	5 (29%)	11 (39%)	0.50
Permanent neurologic deficit	2 (4%)	1 (6%)	1 (4%)	1.00

In the CC cohort, the majority of operations conducted were complete CC, with 70% (N = 19) of patients undergoing complete callosotomy versus 30% (N = 8) undergoing anterior two‐thirds callosotomy. Three patients (11%) underwent the completion of a prior open anterior two‐thirds callosotomy using an open CC approach. In comparison, CCA was performed for complete callosotomy in 78% (N = 7) of patients versus 22% (N = 2) for an anterior two‐thirds callosotomy. Of all CCA procedures (N = 17), seven were performed for disconnection of residual corpus callosum following prior complete CCA (N = 4) or prior open complete CC (N = 3). Additionally, one CCA was performed for the completion of a prior open anterior 2/3 CC (Figure 1). In the CC cohort, residual disconnection was required most frequently for white matter in the genu and splenium (Table 4). This was similar in the CCA cohort, but was more often genu or splenium‐only. The pattern of residual fibers was not statistically different between the groups, although this comparison is limited given the small sample sizes (*P* = 0.49).

**TABLE 4:** Residual CCA white matter ablation.

	Total N = 7	First intervention CCA N = 4	First intervention CC N = 3	*P*‐value
Anatomy of residual				
Genu only	1 (14%)	1 (25%)	0 (0%)	0.49
Splenium only	1 (14%)	1 (25%)	0 (0%)	
Body only	0 (0%)	0 (0%)	0 (0%)	
Genu & splenium	4 (57%)	1 (25%)	3 (100%)	
Genu, splenium & body	1 (14%)	1 (25%)	0 (0%)	

## 4 DISCUSSION

**FIGURE 2:**
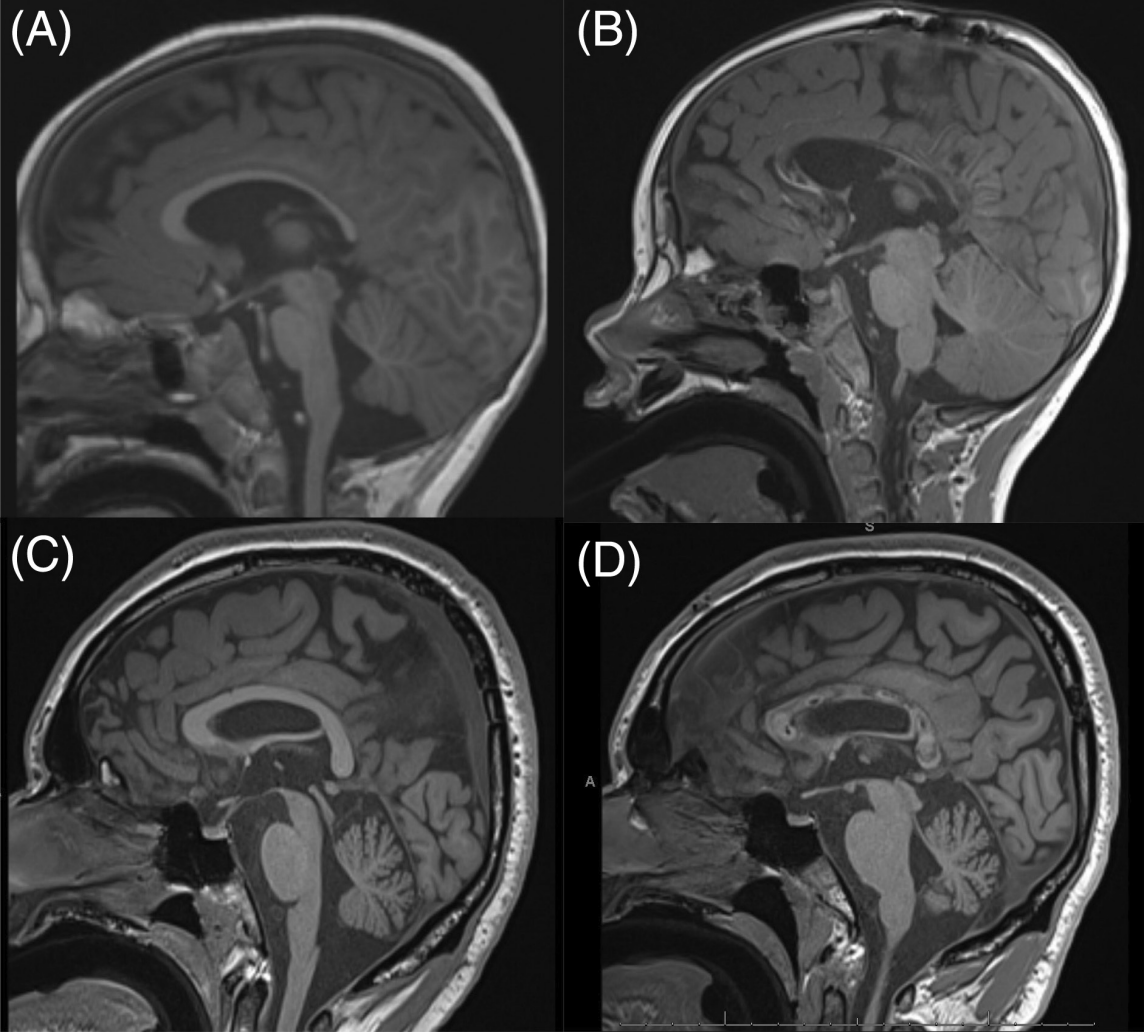
T1‐weighted MRI demonstrated pre‐ and postoperative imaging of patients undergoing open callosotomy (Panels A and B) and corpus callosum ablation (Panels C and D).

Here we present a longitudinal single‐institution experience of performing CCA, a novel minimally invasive approach to treating DRE, while comparing outcomes of CCA to traditional CC with extended follow‐up (Figure 2). Given the novelty of this technique, our findings are an important demonstration of CCA's durability and safety in direct comparison to the traditional open operation.

MR‐guided LITT, first described for the treatment of intracranial metastasis in a 2008 pilot study by Carpentier et al, has garnered widespread adoption for a spectrum of neurosurgical disorders given its relative effectiveness, low complication profile, and decreased postoperative hospital stays.^10^ The 2020 prospective multicenter study by Landazuri et al demonstrated the safety and effectiveness of laser ablation for the treatment of DRE. In this cohort of 60 patients undergoing laser ablation for DRE, 63% of patients achieved seizure freedom at 1‐year follow‐up postoperatively, signaling the promise of LITT for the treatment of epilepsy.^11^, ^12^

Several cohort studies, including a 2022 single‐institution early experience by Mallela et al. at our institution, introduced the potential application of LITT to CC, a palliative disconnective operation for the treatment of atonic seizures.^13^, ^14^, ^15^ In this study of 10 patients undergoing CCA with at least a 3‐month follow‐up, 71% experienced the eradication of atonic seizures, and 57% of patients demonstrated improvement in other seizure types postoperatively.^5^ After 1 year of follow‐up, our study demonstrates 40% of patients undergoing CCA sustained >50% reduction in atonic seizures, suggesting a decline in effectiveness over time that is commensurate with trends seen across pediatric epilepsy surgery. In one large study of long‐term seizure outcomes following CC, Tellèz‐Zenteno et al reported seizure freedom in only 35% of patients undergoing CC 5‐years postoperatively, signaling the palliative role of CC with waning effectiveness over time.^15^ The rates of seizure freedom seen across both cohorts in our series and the aforementioned study further emphasize the palliative role of corpus callosal interventions given the strikingly low percentage (20%) of patients achieving Engle Class I or II at last follow‐up when assessing both cohorts individually and when encompassing the entire study cohort. However, it is worth noting that the number of ASMs used in the CCA cohort was decreased or unchanged in 80% of patients at 1‐year and 83% at last follow‐up, suggesting some benefit to decreasing medication burden, albeit with a diminishing effect over time. Ultimately, it must not be forgotten that the palliative goals of surgery are to diminish bi‐hemispheric seizure propagation, not to eradicate ictal activity.^1^, ^2^, ^16^, ^17^

The apparent waning postoperative effect seen in epilepsy surgery has also been observed across epilepsy surgical techniques, as demonstrated by the meta‐analyses performed by Widjaja et al. and Harris et al in 2020 and 2022, respectively; they demonstrated a decrease in rates of seizure freedom over time in patients undergoing a wide range of open‐resective epilepsy surgery.^18^, ^19^ These outcomes illustrate lower rates of meaningful atonic seizure reduction in CCA compared to the long‐term durability of patients undergoing CC. For instance, in a single‐institution retrospective study on long‐term seizure outcomes after CC, Tanriverdi et al demonstrated 77% atonic seizure improvement at 1‐year follow‐up in their cohort of 95 patients.^20^ These findings are similar to the long‐term target seizure reduction observed in our CC series, with 73% showing improvement in atonic seizures, although further longitudinal studies are warranted to further assess this relationship over time.

Not surprisingly, dissection of the corpus callosum is an effective palliative measure to quell bilateral synchrony of seizure generalization, extending its utility beyond solely reducing atonic seizures. In 2017, Luat et al. described their endoscopic CC series of 20 patients and noted that 67% of patients experienced a greater than 50% post‐callosotomy reduction in GTC seizures.^21^ Similarly, Nei et al demonstrated 79.5% of patients experienced a greater than 50% reduction in GTCs following CC. In the same study of 53 patients undergoing CC, 77.8% of patients were found to have a greater than 50% reduction of either atonic or tonic seizures, underscoring its effectiveness in palliating the spectrum of generalized seizures.^22^ While there was no significant difference in GTC or tonic seizure reduction between the CCA and CC cohorts, we too found meaningful seizure improvement across generalized seizures types in our patient population, regardless of group. Importantly, this effect also appears durable in both cohorts and highlights the utility of callosotomy for a patient population often plagued by multiple seizure types.

Although the disconnection of established seizure networks can result in seizure reduction, few studies have observed an increase in seizure frequency following callosotomy, especially in those patients with focal seizures.^21^ While post‐callosotomy regression in seizure control was rarely observed in our series, it most commonly presented as a transient seizure frequency increase in the first 30 days following surgery and, in some cases, resolved in later outpatient follow‐up. The dynamic nature of epilepsy limits our ability to draw many conclusions from this observation; however, seizure worsening following epilepsy surgery is a known phenomenon commonly attributed to the literature surrounding epilepsy resection surgery, which postulates multiple seizure foci as a primary predictor for this undesirable postoperative outcome.^23^ Similarly, it is possible that disconnection of the “primary” seizure network via callosal disconnection may result in a predominance of new or preexisting seizure networks, resulting in more seizures. While more studies are required to understand the pathophysiology of this observation, our preliminary experience suggests this occurrence is rare and is not associated with either the CC or CCA technique.

Furthermore, the seizure outcomes observed in the CCA may be explained by the large proportion of residual callosotomies performed in this cohort. While this observation highlights CCA's potential for completion of CC without the heightened risk of re‐do craniotomy or posterior interhemispheric approach, in the case of residual splenial fibers, its effectiveness in treating refractory seizures following CCA for callosotomy completion is currently undefined. In a 2019 study by Huang et al of six patients investigating the role of CCA for completion of callosotomy, none of the three pediatric patients treated reported good seizure outcomes at last follow‐up, possibly indicating an underlying seizure mechanism refractory to callosotomy.^8^ Similarly, completion callosotomy via open craniotomy has been demonstrated in the literature with varying results.^24^ However, the rate of inadvertently incomplete callosotomy in the CC or CCA cohort is relatively unknown. Our study not only calls attention to the feasibility of completion callosotomy via CCA but also describes our rates of re‐do CCA for incomplete callosotomy following prior CCA (N = 4/9, 44%) or prior open CC (N = 3/27, 11%). We also show that the genu and splenium were the most common locations to require secondary callosotomy completion. A continued evaluation of de novo CCA rates of complete callosotomy is required to assess its accuracy and effectiveness.

Our study demonstrates a significantly shorter length of stay in patients undergoing CCA compared to their CC counterparts, with a nearly threefold decrease in hospitalization duration. This finding is supported by a 2022 matched noninferiority multicenter study in which 185 patients undergoing LITT were matched to open surgical patients for the treatment of DRE. In this study, the average length of stay was significantly shorter (3.1 days vs 7.2 days) for patients undergoing LITT. Decreased postoperative pain is likely a major contributor to this observation and highlights a fundamental strength of LITT due to its minimally invasive approach. Yossofzai's study also demonstrated a significant decrease in postoperative complications, which invariably contributed to decreased hospitalizations and more discharges home as opposed to rehabilitation centers and potential delays awaiting placement, as well as fewer downstream complications associated with prolonged hospitalizations.^25^ While there was no significant difference in complication rates in our study, the CCA cohort experienced fewer complications (12% vs 28%), mirroring previously published data promoting LITT.^6^, ^26^, ^27^

### 4.1 Limitations

There are several limitations to this study, including its design as a retrospective cohort study at a single institution, which may reduce its generalizability. Additionally, without a standard randomization process, the risk of the introduction of selection bias could impact the surgical intervention offered, preoperative patient expectations regarding the expected duration of hospitalization, and subsequent postoperative management decisions. There is also a risk of timing bias considering the CCA patients were concentrated in the later part of the study's duration, and changes could have resulted in general changes to practice patterns or management strategies. For example, the significant difference in rates of prior VNS implantation between cohorts is presumably secondary to an increased likelihood to offer CCA as a first‐line treatment given its minimally invasive approach combined with favorable preliminary data. Additionally, the retrospective design of this study resulted in discrepancies between the two cohorts, including their baseline demographics, underlying diagnosis, preoperative surgical histories, and extent of disconnection, which invariably complicates the direct comparison of both techniques. Furthermore, the adoption of CCA is relatively new, and as a consequence, the average length of follow‐up is relatively short and the patient cohort is small. Future studies establishing the outcomes after medium, and long‐term follow‐up are necessary. Last, the small sample size precludes the construction of any definitive conclusions. A large‐scale, multicenter, randomized control trial is necessary to contextualize the role of CCA in the treatment of atonic seizures.

## 5 CONCLUSIONS

CCA is a novel, minimally invasive technique that is safe and effective in the treatment of DRE, particular atonic seizures. When compared to traditional CC, CCA provides a similar seizure reduction and is associated with shorter hospital stays. However, results should be interpreted with caution given the small sample sizes. Additional larger‐scale, multicenter trials are necessary to define the role of CCA in the treatment of DRE.

## AUTHOR CONTRIBUTIONS

H.W.P. and T.A. designed the study. J.L.H., E.P., and E.H. coordinated the clinical database and collected clinical data. J.L.H. and T.A. performed data analysis. H.W.P., T.A., and J.L.H. wrote the manuscript. All authors read and commented on the manuscript before submission.

## 6 CONFLICT OF INTEREST STATEMENT

T.J.A. is a consultant for Monteris Medical and receives researching funding as a Site‐PI for Monteris Medical's LAANTERN trial. The remaining authors declare no competing interests.

## ETHICS STATEMENT

We confirm that we have read the Journal's position on issues involved in ethical publication and affirm that this report is consistent with those guidelines.

## Supporting information


Table S1.
