# Supplementary material for: Comparison of magnetic resonance‐guided laser interstitial thermal therapy corpus callosum ablation to open microsurgical corpus callosotomy: A single‐center retrospective cohort study
Source: Epilepsia Open. 2023 Nov 10;9(1):96–105. doi: 10.1002/epi4.12835 (PMC10839368; doi:10.1002/epi4.12835)
Supplement: Supplementary file 1 — Table S1. [file EPI4-9-96-s001.docx]

| **Pt** | **Sex** | **Age @ Sz Onset, y** | **Age @ CC, y** | **Approach** | **Extent** | **Complications** | **Future Surgery** | **F/U, y** | **Engel Score^a^** | **Atonic Outcome^a^** | **GTC Outcome^a^** | **ASM Outcome^a^** |
| --- | --- | --- | --- | --- | --- | --- | --- | --- | --- | --- | --- | --- |
| 1 | F | 0.25 | 10 | CC | Complete | NDA | 0 | 19.9 | 4 | NA | No change | NDA |
| 2 | F | NDA | 11 | CC | Complete | 0 | 0 | 14.7 | 3 | Resolved | NA | No change |
| 3 | M | 2 | 8 | CC | Complete | 0 | 0 | 8.6 | 3 | Decrease | Decrease | Decrease |
| 4 | M | NDA | 11 | CC | Complete | 0 | 0 | 8.6 | 3 | Resolved | NA | Increase |
| 5 | F | 7 | 12 | CC | Anterior 2/3 | 0 | 0 | 7.9 | 4 | No change | No change | Increase |
| 6 | F | 0 | 14 | CC | Complete | 0 | 0 | 6.1 | 4 | No change | No change | Increase |
| 7 | F | 9 | 13 | CC | Complete | 0 | 0 | 5.9 | 3 | Decrease | NA | NDA |
| 8 | F | 2 | 19 | CC | Anterior 2/3 | 0 | 0 | 5.5 | 4 | Decrease | No change | Decrease |
| 9 | M | 0.83 | 12 | CC | Complete | IVH | 0 | 5.3 | 4 | NA | Increase | Increase |
| 10 | F | 2 | 7 | CC | Anterior 2/3 | 0 | VNS | 5.1 | 4 | No change | Decrease | No change |
| 11 | M | 0.66 | 9 | CC | Complete | 0 | 0 | 4.7 | 1 | Resolved | NA | Increase |
| 12 | M | 0.54 | 16 | CC | Complete | 0 | 0 | 3.8 | 3 | Decrease | No change | Increase |
| 13 | M | 1.50 | 6 | CC | Complete | 0 | 0 | 3.8 | 4 | No change | NA | Increase |
| 14 | F | 0.50 | 17 | CC | Complete | 0 | 0 | 3.6 | 3 | Decrease | NA | Increase |
| 15 | F | NDA | 20 | CC | Complete | 0 | 0 | 3.1 | 3 | Decrease | No change | NDA |
| 16 | F | 0.50 | 2 | CC | Complete | 0 | 0 | 2.7 | 1 | NA | NA | Decrease |
| 17 | M | 7 | 17 | CC | Complete | Hemorrhage | 0 | 2.4 | 2 | Resolved | Decrease | Increase |
| 18 | M | 1 | 14 | CC | Anterior 2/3 | 0 | VNS removal^b^ | 2.0 | 4 | No change | No change | No change |
|  |  |  | 15 | CC | Completion^b^ | 0 |  |  |  |  |  |  |
| 19 | M | 8 | 10 | CCA | Anterior 2/3 | 0 | VNS | 1.8 | 4 | No change | No change | No change |
| 20 | F | 0.42 | 13 | CCA | Complete^b^ | 0 | VNS^b^ | 1.3 | 2 | Resolved | NA | No change |
| 21 | M | 2 | 13 | CC | Anterior 2/3^b^ | IVH | VNS^b^ | 1.3 | 2 | Resolved | No change | No change |
|  |  |  | 15 | CCA | Completion | 0 |  |  |  |  |  |  |
| 22^c^ | F | 0.42 | 7 | CC | Partial | Hemorrhage | 0 | 1.2 | 3 | Decrease | NA | Decrease |
|  |  |  | 8 | CC | Completion | 0 |  |  |  |  |  |  |
| 23 | M | 0.25 | 21 | CCA | Complete^b^ | IVH | VNS^b^ | 1.2 | 3 | Resolved | Decrease | No change |
| 24 | M | 0 | 3 | CC | Complete^b^ | EDH, IVH | VNS^b^ | 1.1 | 4 | No change | NA | Increase |
|  |  |  | 4 | CCA | Residual | 0 | HS |  |  |  |  |  |
| 25 | F | 12 | 18 | CC | Anterior 2/3 | EDH | RNS^b^ | 0.8 | 4 | No change | Decrease | No change |
|  |  |  | 19 | CCA | Completion | 0 |  |  |  |  |  |  |
|  |  |  | 20 | CCA | Residual^b^ | 0 |  |  |  |  |  |  |
| 26 | F | 2 | 6 | CCA | Complete^b^ | 0 | VNS^b^ | 0.6 | 4 | Resolved | NA | Decrease |
|  |  |  | 8 | CCA | Residual | 0 | RNS |  |  |  |  |  |
| 27 | M | 0.58 | 4 | CC | Anterior 2/3 | 0 | Resection^b^ | 0.5 | NDA | NDA | NDA | NDA |
|  |  |  | 19 | CC | Completion^b^ | Abscess, infection, CSF leak | VNS |  |  |  |  |  |
| 28 | M | NDA | 51 | CC | Complete | 0 | NDA | 0.3 | NA | Resolved | NA | NDA |
| 29 | M | 15 | 16 | CCA | Complete | 0 | 0 | 0.3 | NA | Decrease | No change | No change |
| 30 | F | 3 | 17 | CCA | Complete | EDH | 0 | 0.2 | NA | NA | Decrease | No change |
|  |  |  | 18 | CCA | Residual | 0 |  |  |  |  |  |  |
| 31 | M | 5.5 | 19 | CCA | Anterior 2/3 | 0 | 0 | 0.1 | NA | No change | NA | No change |
| 32 | F | 0.25 | 10 | CCA | Complete | 0 | 0 | 0.1 | NA | Resolved | NA | Increase |
| 33 | M | 6 | 16 | CC | Complete | 0 | 0 | 0.1 | NA | Decrease | Decrease | No change |
| 34 | F | 0.08 | 2 | CC | Complete | IVH | 0 | 0.1 | NA | NA | NA | No change |
| 35 | M | 4 | 10 | CC | Complete | EDH | 0 | 0 | NDA | NDA | NDA | NDA |
|  |  |  | 15 | CCA | Residual | 0 |  |  |  |  |  |  |
| 36 | F | 1 | 9 | CCA | Complete | 0 | VNS^b^ | 0 | NDA | NDA | NDA | NDA |
|  |  |  | 10 | CCA | Residual^b^ | 0 |  |  |  |  |  |  |

a=Calculated at longest follow-up from latest procedure; b=Procedures complete within same operation; c=No follow-up from latest procedure, outcomes reported based on first procedure

NDA=No data available; NA=Not applicable; IVH=Intraventricular hemorrhage; VNS=Vagus nerve stimulator; EDH=Epidural hematoma; HS=Hemispherotomy; RNS=Responsive neurostimulator
